# Are fishery management upgrades worth the cost?

**DOI:** 10.1371/journal.pone.0204258

**Published:** 2018-09-20

**Authors:** Tracey Mangin, Christopher Costello, James Anderson, Ragnar Arnason, Matthew Elliott, Steve D. Gaines, Ray Hilborn, Emily Peterson, Rashid Sumaila

**Affiliations:** 1 Bren School of Environmental Science & Management, University of California, Santa Barbara, CA, United States of America; 2 Sustainable Fisheries Group, Bren School of Environmental Science & Management, University of California, Santa Barbara, California, United States of America; 3 Institute for Sustainable Food Systems, University of Florida, Gainesville, Florida, United States of America; 4 Department of Economics, University of Iceland, Reykjavik, Iceland; 5 California Environmental Associates, San Francisco, California, United States of America; 6 School of Aquatic and Fishery Sciences, College of the Environment, University of Washington, Seattle, Washington, United States of America; 7 Fisheries Economics Research Unit, University of British Columbia, Vancouver, British Columbia, Canada; Aristotle University of Thessaloniki, GREECE

## Abstract

Many analyses of fishery recovery have demonstrated the potential biological and economic benefits of management reform, but few have compared these to the associated costs of management upgrades, which can be substantial. This study aims to determine if the projected economic benefits of management reform outweigh the increases in management costs required to achieve those benefits. To answer this question, we developed a database of country-level fisheries management costs and use those to estimate the country-level costs of management changes. We use this framework to compare estimates of future costs of management upgrades against their economic benefits in terms of profit. Results indicate that for most nations, including the top 25 fishing nations, management upgrades outweigh their associated costs. This result is robust to a number of alternative assumptions about costs. Results also suggest that stronger reforms such as rights-based management, although sometimes more expensive to implement, can lead to greater net economic benefits compared to alternatives.

## Introduction

While analyses of fishery recovery have demonstrated the potential biological, economic, and social benefits of management reform, few studies incorporate the costs associated with the implementation and maintenance of these reforms. Sustained overfishing leads to population decline, which results in a less productive fishery in terms of both harvest and profit. Management that effectively controls fishing effort can prevent fishery decline or recover depleted stocks, resulting in greater long-term harvest and profits. However, available data suggest that the cost of fishery management is often substantial and that additional costs from major upgrades in management could be prohibitive in some countries. The theoretical implications of fishery management costs for optimal fisheries policies have been investigated by others [1–3]. But to our knowledge a geographically extensive empirically-grounded study comparing the country-level benefits of fishery management improvements to the additional costs of designing and implementing these reforms has never been undertaken. In this study, we estimate the costs of fishery management upgrades and examine whether undertaking these reforms is economically justified.

Conducting a geographically comprehensive comparison of the benefits and costs of fishery management reform requires combining large datasets with new models, and is fraught with uncertainty. While the benefits of reform have been well-documented, the incremental management costs required to achieve those benefits have received very little attention, and no systematic database exists with global coverage. Thus, it remains an open question whether the benefits exceed the costs.

As a starting point, we suggest the following back-of-the-envelope calculation that suggests that for most countries, benefits do indeed clearly outweigh the costs. Using a recent dataset of the economic upside from fishery management reform at the country level [4], we find that the median increase in value per ton of landings that arises from fisheries that undergo management upgrades is about USD 1,515. This is over 15 times Sumaila et al.’s [5] estimate of global management cost per MT in 2003 (USD 97). The rest of this paper is devoted to more specifically estimating the benefits and costs of current management and future upgrades in management, ultimately resulting in a country-level benefit-cost ratio for management improvements.

With this simple calculation as a backdrop, this study has three objectives: 1) to estimate the current cost of managing commercial fisheries in the world’s major fishing countries; 2) to estimate the concomitant change in cost under a suite of alternative management approaches; and 3) to compare these costs with recent estimates of the economic benefits of fishery reform. The work is primarily practical–our goal is to derive ballpark estimates of these values to answer the question whether the potential benefits commonly justify the likely increase in management costs.

Our estimates are admittedly imprecise. They represent a first attempt to estimate important factors needed to determine what constitutes optimal fisheries management. We hope this study will motivate future research on the cost of fisheries management reform in particular countries or fisheries.

## Review of the literature

We adopt the definition of “management costs” used by Schrank et al. [6], which includes the following set of fishery management activities: 1) administration (or management), 2) research, and 3) surveillance and enforcement. Administrative services include administration activities such as monitoring licenses and permits, and adjusting management settings such as an annual total allowable catch (TAC). Research services generate information about the fishery, which is used to inform the design and implementation of fishery management systems and regulations, such as an appropriate TAC, gear restrictions, and closures. Examples of research services include stock assessments, biological and economic data collection, and analysis conducted by the fishing industry and/or institutions responsible for informing or conducting management activities. Finally, surveillance and enforcement services include activities that monitor the fishery and enforce relevant regulations. An at-sea example of enforcement services includes patrolling with vessels, airplanes, or onboard observers. Fisheries enforcement also takes place on land, for example, when officers assess the volume of landed catch and inspect catch composition, vessels, and fishing gear at landing ports, as well as in the assessment of guilt and sanctions and the associated legal proceedings.

Although data on fishery management costs are limited, there have been a handful of useful studies on the topic. Existing studies suggest that, while extremely heterogeneous across countries, the cost of fishery management can be quite substantial [6–9]. Sumaila et al. [5] characterize the three management services, among others, as beneficial subsidies, which they found amounted to USD 7.94 billion globally in 2003, or about USD 97 per MT [5,10]. The majority of these subsidies (USD 5.16 billion) were spent in developed nations–we also find that developed nations tend to spend more on fisheries management services than developing nations. A detailed study of management costs in Newfoundland, Iceland, and Norway revealed that annual management costs in the 1990s in each region ranged between 3 and 28% of the value of landings [7]. Wallis and Flaaten’s analysis of 26 OECD countries in 1999 found that, on average, countries spent 6% of the value of landings on management costs. However, there was a substantial range across countries, with costs ranging from 0 to 70% of landed value [9]. Studies focused on specific developed nations have found that management costs for countries such as Australia, the UK, and the United States have accounted for 7–30% of landed value [11]. Other studies reveal that annual management costs for Thailand ranged between 0.7% and 1.64% of landed value from 1991–1999 [6] and costs in Namibia ranged between 3.7% and 5.9% of landed value over the five-year period of 1994–1999 [12].

Arnason et al. [3, 7] also examined the costs associated with each of the three management services and found that while these costs vary significantly among countries in absolute terms, the relative size of these components were similar in Iceland, Newfoundland, and Norway. On average, enforcement services were the most expensive, representing 59% of management costs, followed by research services at 34%, and finally administration services, representing 7%. Costs associated with surveillance and enforcement services are typically the most expensive, because they are labor intensive and require expensive equipment [3, 7, 8].

While these previous studies of fisheries management costs provide estimates of management cost as a fraction of revenue, they neither provide global coverage of costs, nor, more importantly in the current context, a functional relationship between management costs and the various alternative management regimes. Here, we make, to our knowledge, a first attempt at estimating the potential costs of fishery management upgrades at the country level.

## Materials and methods

### Materials

Conducting a geographically comprehensive analysis of the benefits and costs of fishery management reform requires combining several large datasets with new datasets and approaches. The majority of the data used in this analysis are from two datasets: 1) a database of country-level fishery management costs that we developed for this study; and 2) a global database of annual fishery-level information and indicators, including harvest and profit over time. The second database includes historical values as well as projected future values under different management scenarios, determined using a bioeconomic modeling approach [4]. Each dataset compiles information from other sources.

The database of country-level fishery management costs includes reported expenditures on administration, research, and enforcement services. We collected this information from the OECD’s Government financial transfers database [13], Fishery Ministry reports, and, in one case, a personal communication. The OECD’s database includes information regarding (national and, where relevant, European Union) government financial transfers to the fishing sector for the three management categories. These values were collected by the OECD’s Fisheries Committee (COFI) on an annual basis between 2000 and 2015 from institutions (e.g., Fisheries Ministries, National Statistics Offices) in participating countries using a standard database questionnaire. The relevant institutions report total government expenditures on administrative, research, and enforcement services. Similarly, the Fishery Ministry reports and personal communication (representative from a Fishery Ministry) from which we collected information provide cost values for some or all of those three categories. Management cost values that were not reported in USD are converted using the World Bank’s official exchange rate data, and, where appropriate, reported costs are inflated/deflated into 2012 US dollars using the U.S. Bureau of Labor Statistics consumer price index.

The global fishery database was developed by a previous study and contains information on individual fisheries around the globe representing 78% of reported global harvest [4]. This database includes fishery-specific information on current and projected future biomass, harvest, and profit indicators as well as current management. Future values were projected using a bioeconomic modeling approach that pairs the Pella-Tomlinson surplus projection model [14] with an economic model [4] under different management scenarios. In this study we use projected future harvest and profit indicators under three management scenarios as inputs in our analysis (see Methods for more details).

### Methods

Our approach involves the following five steps: 1) Estimate the current cost of management at the country level; 2) determine the current management approaches in each country by categorizing current landings by management category; 3) develop a model of incremental costs by coupling country-level cost and management data with survey results regarding the relative cost of different management approaches; 4) apply the model to determine the costs of management under different management scenarios; and 5) evaluate management reform options by comparing the difference in anticipated profits (extracted from a recent paper [4]) and anticipated management costs under alternative management interventions and assumptions.

#### Estimate the current cost of management at the country level

To complete this step, we first compile a management cost database (S1 File) from existing data. Our study follows the tradition of earlier work by defining management costs as expenditures on administration, research, and enforcement services. For each country, we collect the annual costs of administration, research, and enforcement services in every year for which data are available. These estimates represent total reported expenditures, which may include funding from the fishing industry. For example, the fishing industry is responsible for many management expenditures in Australia–these funds are collected by the government via a levy or fee [15]. Since we focus on national reports, the data for some countries such as the United States include only information on federally managed fisheries. In addition, while we focus on the commercial fishing sector, it is likely that these expenditures are also representative of some costs associated with managing the recreational sector, as both may benefit from the same management services. Only countries for which we are able to find values for at least two out of the three management cost categories are included in the cost database. Using government reports and statistics, as well as the OECD’s Fisheries Database [dataset 13], 21 countries with values for all three types of management costs are identified. We then filter our database for the most recent data and define these as “current cost” values. While we focus on the most recent data available throughout the rest of this analysis, we also repeat the entire study using the average of all cost entries for each management category to account for the fact that costs can vary over time. The main results hold under this approach–we present key comparisons in the Supplementary Information (see section A in S2 File, S1 Table, and S1 Fig).

**Fig 1:**
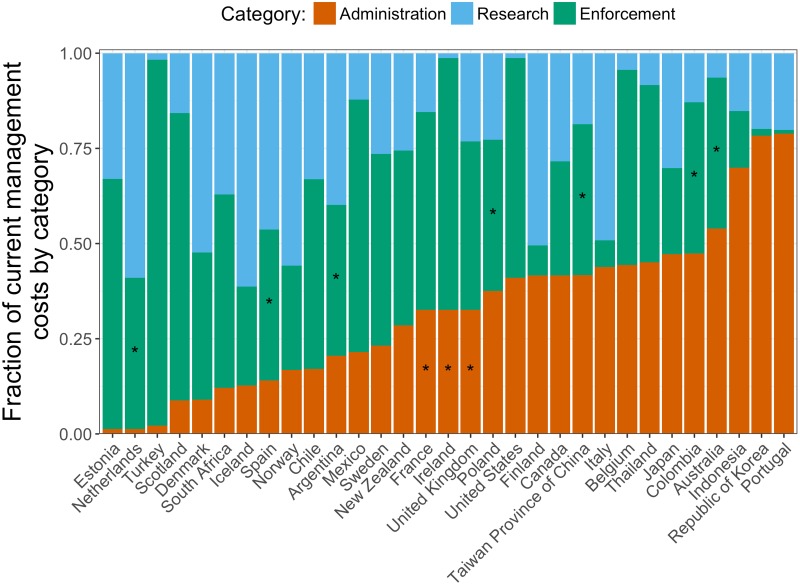
Breakdown of current management costs into management service categories by country. An asterisk (*) represents an estimated cost.

For the 21 countries with values from all three types of management costs, on average, the percentages of total management costs attributed to administration, research, and enforcement services are 32.6%, 27.7%, and 39.7% respectively. Using these averages, we impute the missing cost category (and thus total annual cost of management) for countries for which we are only able to find values from two categories (Fig 1).

**Fig 2:**
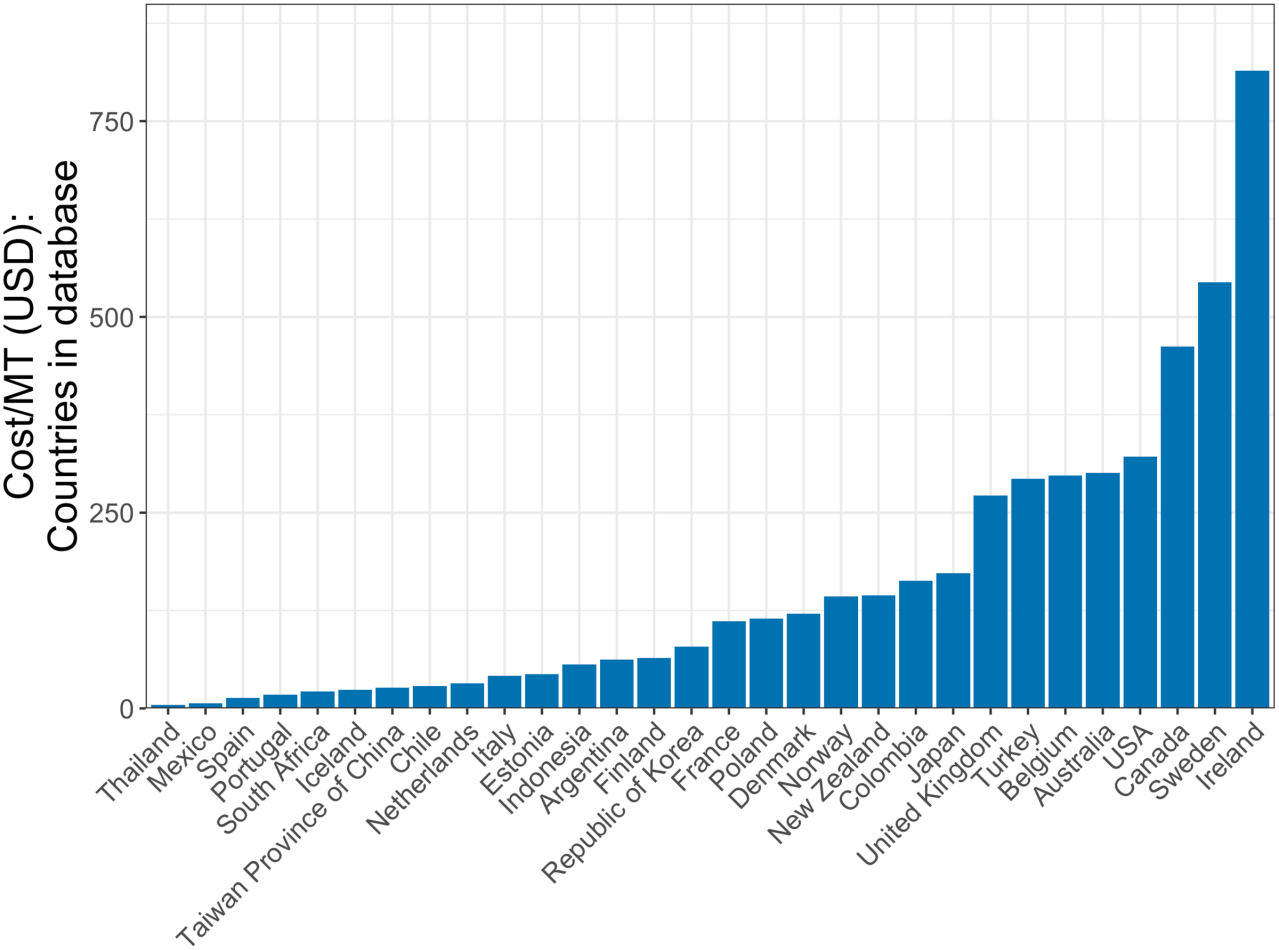
Current cost per MT. Total management cost divided by 2012 landings for the 30 countries included in the management cost database included in this analysis.

Total costs are then scaled to match the percentage of total landings represented in the global fishery database, which contains fishery level data for fisheries around the world [4]. This database covers a percentage of the 2012 FAO reported total landings for each country [16]. A country that has 50% coverage means that the fishery database covers 50% of the total FAO reported landings for that nation. For example, the global fisheries database that we use captures 91.3% of 2012 FAO reported harvest for the United States. On average, the fishery database covers 58% of catch for each country. Therefore, we scale the total cost value to match this percentage. For the remainder of this paper, the term total cost represents the scaled total cost. Finally, we follow the most recent literature and normalize these costs by landings (cost / MT). We determine the cost per MT of landings in each country by dividing the total annual cost of management by 2012 harvests (Fig 2). It may alternatively be reasonable to scale management cost by landed value–while we focus on calculations that scale management costs by landings to match with existing literature and comport with our global databases (which include more data regarding landing volumes compared to revenues), we repeat the analysis scaling by landed value and present relevant findings in the Results section.

We recognize that some costs may be fixed, and thus should not be normalized. However, there is limited information regarding this breakdown across countries. If some portion of management cost is fixed (i.e. it does not scale with catch), then by assuming all costs are variable, we will overestimate the cost of management upgrades, and in that sense our results are conservative (because even in that case, we will find that the increase in cost is small relative to the increase in benefit for most countries).

**Fig 3:**
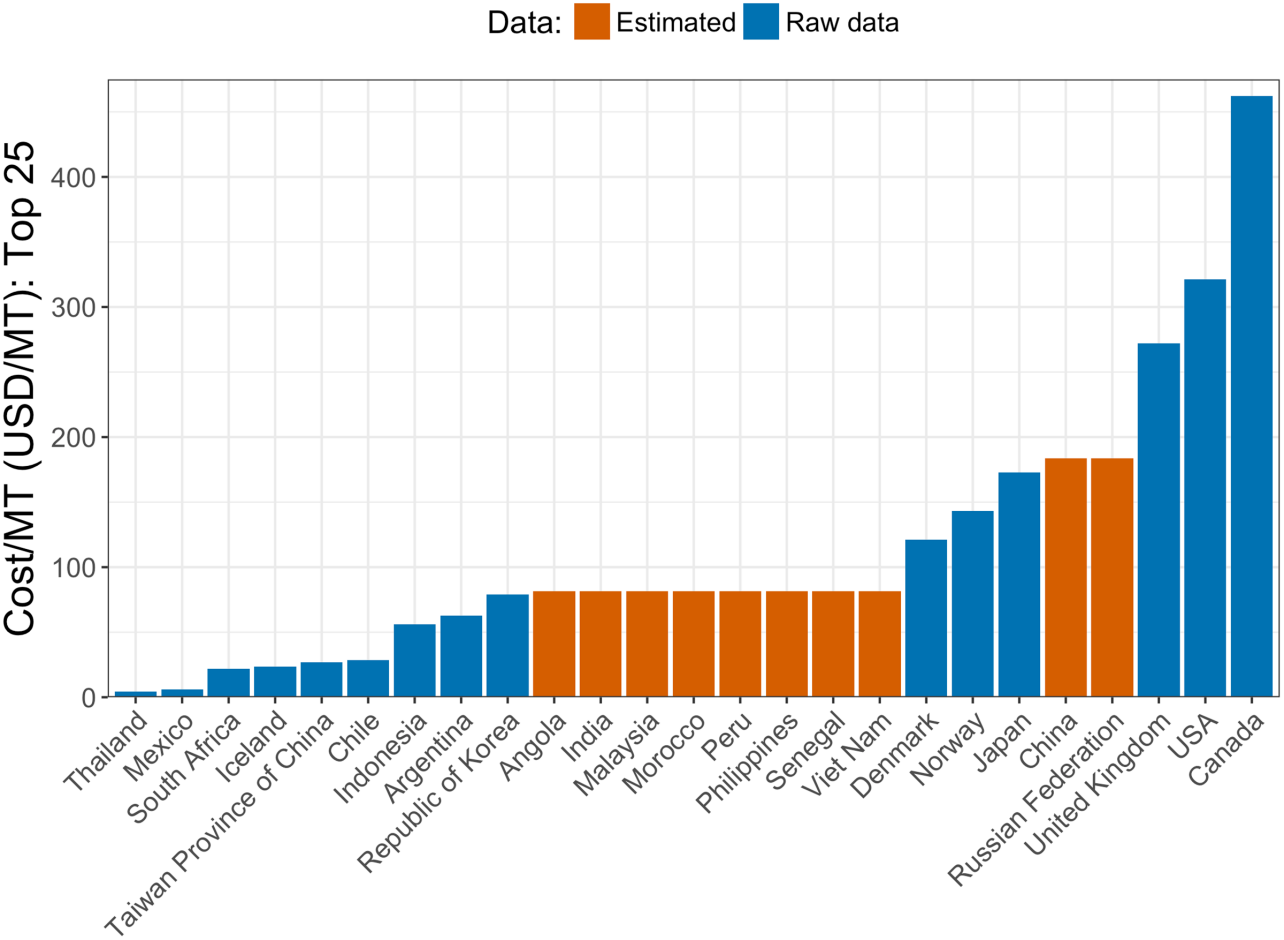
Current cost per MT for top 25 countries in terms of 2012 landing volume. Total management cost divided by landings for the top 25 countries in terms of total volume landed in 2012.

In order to determine the current management cost in fishing countries for which we do not have adequate data, we impute the value from “similar” countries for which we do have data. We follow an approach outlined in a previous study [5], which groups countries according to their Human Development Index (HDI) scores, which measures development based on life expectancy, education attainment, and standard of living. This index was developed by the United Nations, and we use the most recent scores available (2015). We follow Sumaila et al.’s (2010) approach of defining countries with an HDI score ≥ 0.80 as “Group I” and those with an HDI score between 0 and 0.79 as Group II. We make the same country-specific adjustments, and group countries without an HDI score using those of countries in the same geographic area. Next, we calculate the average cost of management (per MT) for each group. In countries for which we lack management cost data, we apply the average from the relevant group: Group I countries missing data are assigned the average from Group I (USD 184 per MT) and Group II countries that are missing data are assigned the average from Group II (USD 81 per MT) (Fig 3). We do this in order to obtain estimates of management costs for major fishing nations that are pertinent to the present study, as well as for the globe. An alternative would have been to parameterize a regression model of management cost as a function of various country-level characteristics (e.g., GDP, GDP per capita). But owing to the stark data limitations, that regression model provided only weak evidence of systematic relationships. Thus, we employ the “Group I” and “Group II” approach described above to impute baseline management costs to countries for which original data are unavailable.

Importantly, we impute cost per MT values for some of the most important fishing nations by volume, including China, India, Peru, Viet Nam, and Russia. Due to the uncertainty of estimated values, we perform an additional analysis in which we make the “extreme” assumption that the cost per MT is nearly 500 USD for all strong management, which provides a very high upper bound on the possible cost from management upgrades.

#### Determine current management approaches in each country by categorizing current landings by broad management categories

To determine how changes in management type will affect management cost, we require information about the current approaches used to manage fisheries in each country. A comprehensive global database of fishery management is not available. To fill this void, we categorize landings in each country into three broad management categories: 1) *Catch share* (CS); 2) Strong *catch controls* (CC); and 3) A broad “other” category, which we refer to loosely as *open access* (OA). To assign management categories to fisheries, we use the global fishery database [4] containing management data derived from information in Environmental Defense Fund’s Catch Share database [17] and the RAM Legacy Stock Assessment Database [18]. The RAM Legacy Stock Database (Version 2.95) contains detailed stock assessment information for 397 stocks, including both fish and invertebrate stocks. The information was collected from over 20 national and international management agencies [18].

**Fig 4:**
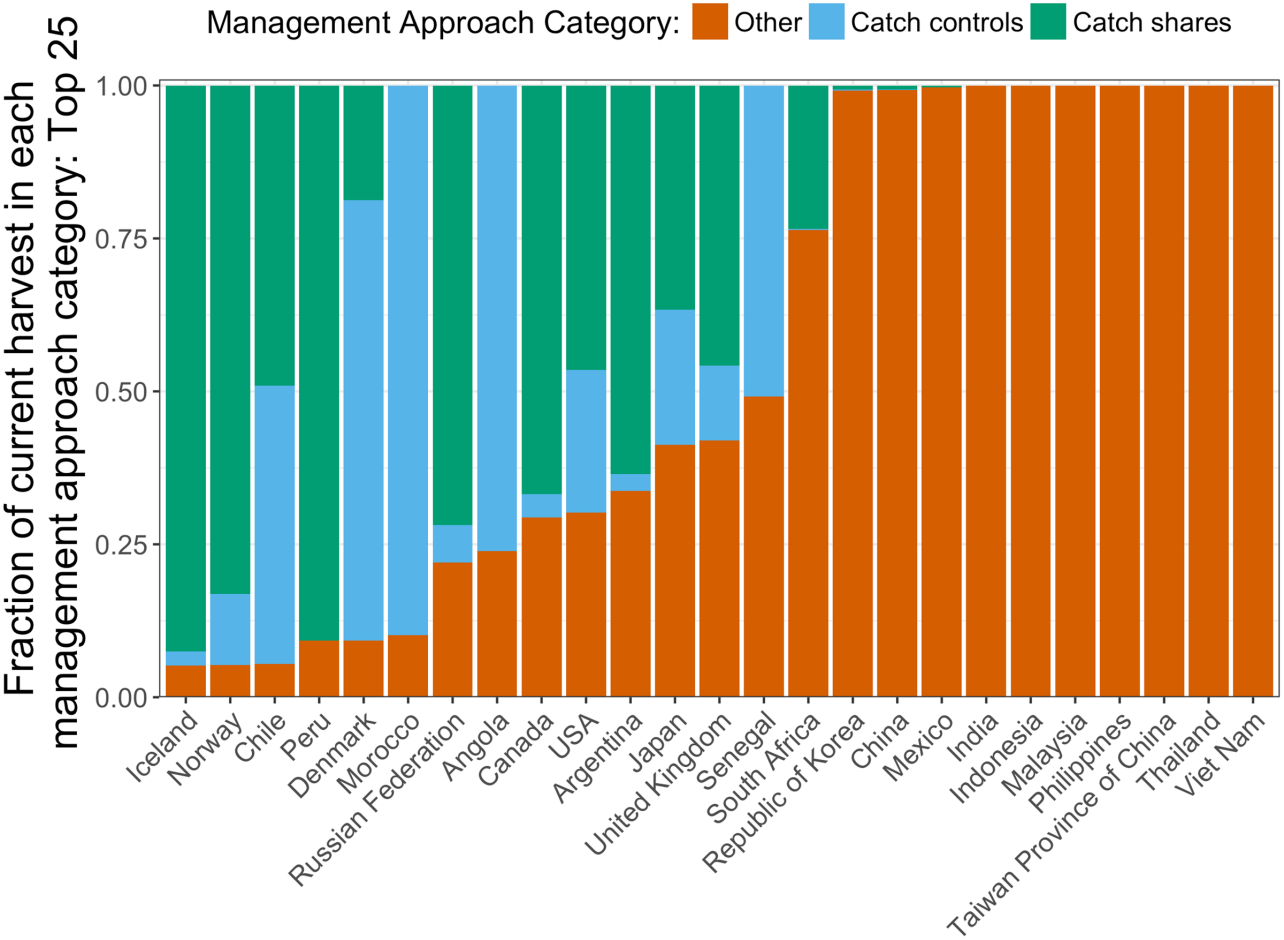
Breakdown of current assessments and approaches to management in the top 25 countries in terms of 2012 total landings. The “Other” category represents landings from fisheries managed under input controls and/or unregulated open access.

Landings from fisheries classified as being managed under a catch share regime (e.g., community-based allocation, individual quotas, individual vessel quotas, individual transferable quota, and TURFs) are counted as CS landings. The landings from fisheries present in the RAM database that are not managed under catch shares are counted as CC landings. Because implementing informed regulations that control harvest requires a stock assessment, we consider fisheries in the RAM database to have strong management. Therefore, the CC category is meant to represent the broad range of management that can be classified as strong biological management without catch shares. Finally, all other fisheries are categorized as OA (Fig 4 and S3 Fig).

We exclude tuna and billfish fisheries from this analysis due to the fact that they are highly migratory species and largely managed by international agencies (RFMOs) and not governments.

#### Develop a model of incremental costs

In order to estimate how management costs could change as a result of various management reforms, we develop a model of the incremental costs of management upgrades. This model is parameterized using survey responses from a group of nine fishery management experts from around the globe. First, we survey a group of twenty experts using a simple pre-questionnaire. These experts, in addition to several authors of this study, were gathered for a meeting on global fishery status and management. Meeting attendees were given the opportunity to participate in the pre-questionnaire, and were informed as to how the information would be used in our study. To collect more specific information, we administer a second more detailed survey (S3 File) to a subset of the experts. These nine respondents are globally representative and were chosen due to their relevant experiences with the topic of fishery management costs. We did not seek approval by an institutional review board (IRB) because our activity falls under the category of “Fact-collecting interviews,” which are questionnaires focused on things, products, or policies rather than personal information. In addition, we included introductions in our questionnaires that clearly described how the information obtained would be used in the current study and ensured anonymity. According to our institution’s Office of Research guidelines, this activity typically does not require an IRB review.

**Fig 5:**
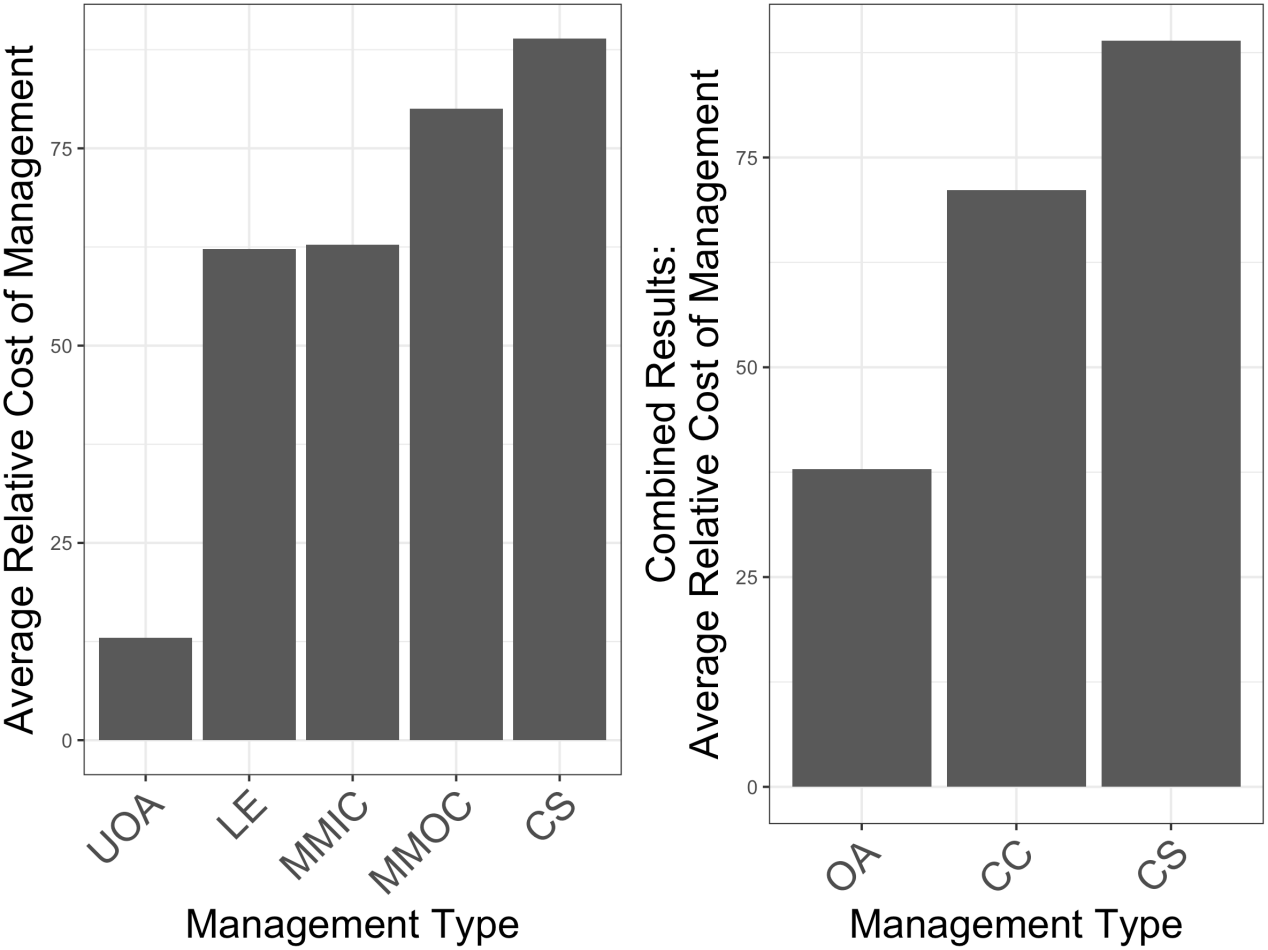
Survey results. Results on the left reflect how respondents rank the relative cost of unregulated open-access (UOA), limited entry (LE), morality management with input controls (MMIC), mortality management with output controls (MMOC), and catch shares (CS). The figure on the right represents the relative cost open access management (OA, achieved by combining results for UOA and LE), management utilizing catch controls (CC, achieved by combining MMIC and MMOC), and catch shares (CS, which remained the same).

We ask these experts (academics and practitioners) to rank five different management types (unregulated open access, limited entry, mortality management with input controls, mortality management with output controls, and catch shares) in order from least expensive to most expensive in terms of actual costs. Then, the respondents are asked to determine the relative cost of each management strategy, assuming that the management strategy ranked as most expensive costs 100. We average scores across respondents (Fig 5, left panel), and present variance in responses in the Supplementary Information (S2 Fig). Finally, we lump management approach categories to create management reform scenarios that match those used to project future profit indicators. This allows us to compare costs and benefits. We combine the values for unregulated open access and limited entry to represent the category “broadly open access” (OA). Next, we combine the values for mortality management with input controls and mortality management with output controls to represent the category strong harvest controls. We call this category *catch controls* (CC) because in practice effective mortality controls may be achieved through carefully designed input or output controls. The results (Fig 5, right panel) suggest that the cheapest category of management contains unregulated open access fisheries and fisheries that use limited entry systems (labeled “OA” in the second panel of Fig 5). The next most expensive category is the group of fishery management approaches that employs direct output controls or input controls (labeled “CC”). This category requires about 80% higher cost than the former category. Survey results suggest that the most expensive category contains approaches with formal catch shares (labeled “CS”)–this category costs about 25% more than the “CC” approach. For the remainder of this analysis, we will use the fishery management categories identified in the second panel of Fig 5.

#### Apply model to determine the future costs of management under different management scenarios at the country level

We develop a simple model to estimate the incremental costs of managing fisheries using OA, CC, and CS approaches in each country. We assume that the aggregate total cost of managing all fisheries in a country will depend on the portion of landings that are managed under each category of management, the relative costs of those management categories, and a country-specific constant that scales the total cost of management.

$$TC_{i}=s_{i}\sum_{j=1}^{3}H_{i,j}c_{j}$$ (1)

Eq 1 shows the total current cost of management in country *i* (*TC*_*i*_) where *H*_*i*,*j*_ is the harvest in management category *j* (we use *j* = 1 to represent OA, *j* = 2 to represent CC, and *j* = 3 to represent CS) in country *i*, *c*_*j*_ is the relative cost of management type *j* (which is the same for all countries), and *s*_*i*_ is a country-specific constant that scales the total cost of management. Eq 1 can be rewritten in terms of cost per MT by using the fractions of total harvest (rather than the volumes) managed under each management category:

$$C_{i}=s_{i}\sum_{j=1}^{3}h_{i,j}c_{j}$$ (2)

where *C*_*i*_ is the current cost per MT in country *i* and *h*_*i*,*j*_ is the fraction country *i*’s total harvest in management category *j*.

The constant *s*_*i*_ is included to capture differences between countries that might affect the cost of and/or the expenditure on fisheries management, such as the different labor and fuel costs among countries. While we assume that the relative cost of each management type is the same across countries (*c*_1_, *c*_2_, and *c*_3_ are the same for each country), we allow the absolute cost of management per MT to vary across countries, as captured by the parameter *s*_*i*_.

**Fig 6:**
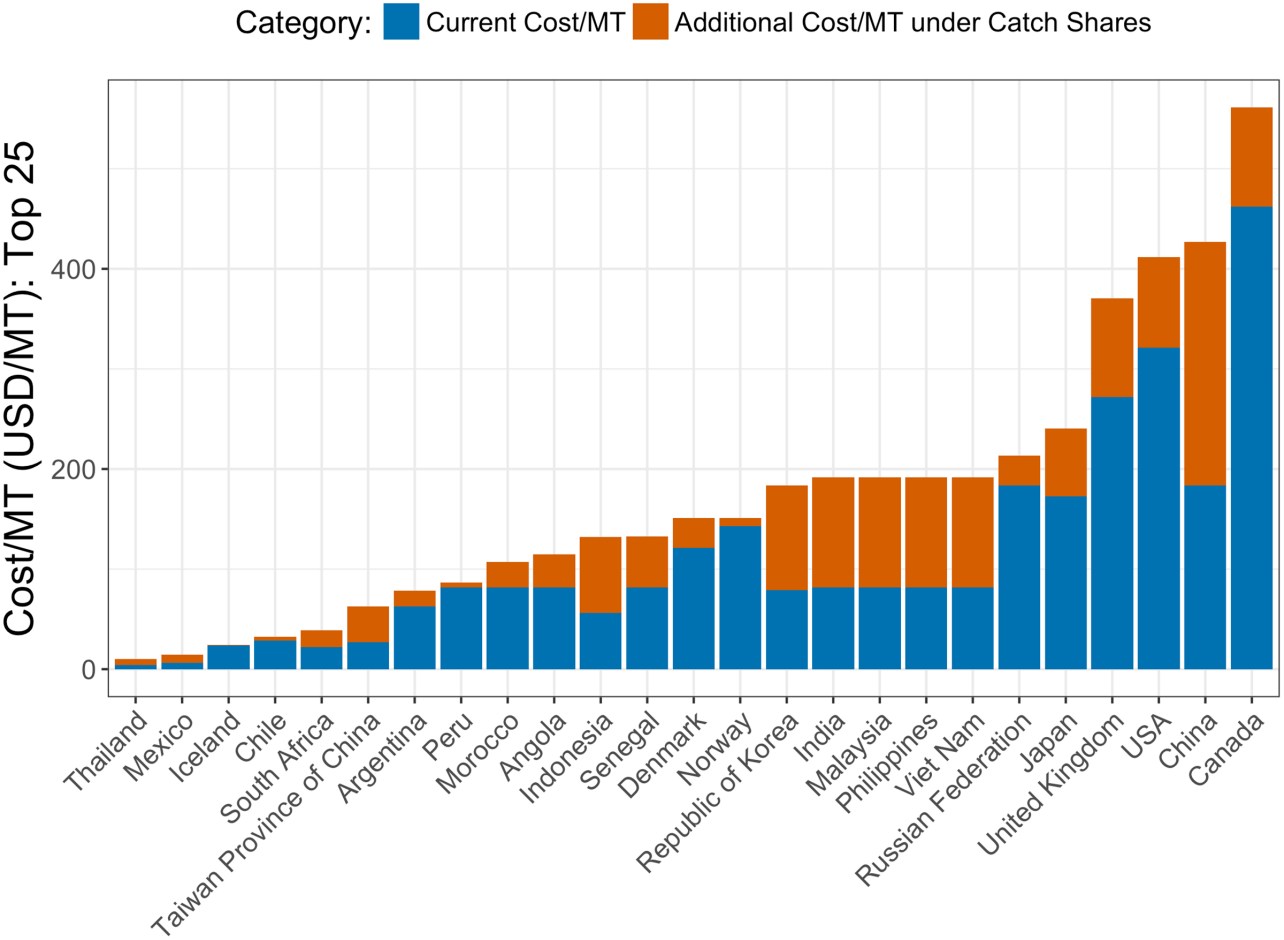
Current cost per MT and anticipated incremental cost under catch shares. These together give the anticipated cost per MT when all fisheries are managed under catch shares (sum of stacked bars). This subset includes the top 25 countries in terms of 2012 landings.

To back out a country’s cost parameter (*s*_*i*_), we re-arrange Eq 2 and solve for *s*_*i*_ using each country’s current cost per MT, the relative costs of management (*c*_*j*_) extracted from the surveys, and harvest data. It can be interpreted as follows: The cost per MT of managing all fisheries in a country under a particular management approach is *s*_*i*_ multiplied by the relative cost of that management approach, *c*_*j*_. For example, the cost per MT of managing all fisheries with catch shares is *s*_*i*_ multiplied by the relative cost of CS management (Fig 6).

Having calculated *s*_*i*_ for each country, it is then possible to calculate the future cost of management in each country. If at some future date, the total harvest in management category *j* is *H*_*i*,*j*_*′* then the total future management cost (*TC*_*i*_’) is simply:

$$TC_{i}\prime =s_{i}\sum_{j=1}^{3}H_{i,j}\prime c_{j}$$ (3)

For example, if catch shares are adopted in all fisheries in a country and the resulting total catch at some future point in time is projected to be *H*_*i*,3_*’*, the new cost of management is simply the following:

$$TC_{i}\prime =s_{i}H_{i,3}\prime c_{3}$$ (4)

Comparing future projections with the current estimated cost provides an estimate of the change in fishery management cost arising from the change in management. Denoting the change in total harvest in management category *j* (resulting from a management change) as Δ*H*_*i*,*j*_, the change in total management cost (Δ*TC*) resulting from a fishery management reform can be written:

$$\Delta TC_{i}=s_{i}\sum_{j=1}^{3}\Delta H_{i,j}c_{j}$$ (5)

The two reform scenarios that we focus on are (1) managing all fisheries that are not already managed by catch shares with strong catch controls (CC Scenario); and (2) managing all fisheries using catch shares (CS Scenario). These will both be compared to a BAU (business as usual) scenario, further described in the following subsection.

An alternative to the incremental cost model described above would be to empirically estimate how a country’s costs depend on the management approach used in that country. If we had a reliable panel data set of management cost across the world’s fisheries, that approach would be ideal. But because data are extremely sparse, we have developed and employed the model above, where the relative costs in each management category (*c*_*j*_) is derived from expert interviews. We believe that this approach results in conservative management cost estimates as it scales all costs linearly to landings. Indeed, we may even expect the marginal cost of management to decrease as stocks rebuild and harvest increases. In that case, the estimates provided here would overestimate future management costs.

#### Evaluate management reform options by comparing difference in profits and management costs under different management interventions

The final step is to determine whether the benefits of a given reform exceed the incremental costs of adopting that reform. In order to evaluate management reform options, we compare the future harvest and profit trajectories under a business as usual (BAU) scenario to the outputs of the two management upgrade scenarios–CS Scenario and CC Scenario (Table 1). The BAU scenario assumes that management upgrades are not adopted. In this scenario, all fisheries remain under the same management approach used currently–fisheries managed by OA, CC, and CS management remain under that form of management. Under the CC scenario, all fisheries currently managed with catch shares remain under CS management, and the rest are managed under CC management. Finally, under the CS scenario, all fisheries are managed with catch shares.

**Table 1:** Management scenario descriptions. Descriptions of fishery management under each of the three future management scenarios.

	Future Fishery Management		
Current Fishery Management	Business as Usual (BAU) Scenario	Catch Control (OC) Scenario	Catch Share (CS) Scenario
Broadly open access (OA)	OA	CC	CS
Catch control (CC)	CC	CC	CS
Catch share (CS)	CS	CS	CS

Future country-level management costs are calculated using projected harvest under each management category in the year 2050 for each of the three management scenarios. These harvest values are extracted from the global fishery-level database developed by Costello et al. (2016). Future management costs under the BAU scenario are calculated for each country by multiplying the modeled 2050 harvest (for each category of management) by the cost per MT, according to Eq 3. We calculate future management cost under the CC Scenario for each country using Eq 3, and substitute the modeled 2050 harvest (extracted from the global fishery database [4]) under the CC Scenario for *H*, and set *H*_*i*,1_’ = 0, *H*_*i*,2_’ = (total 2050 harvest minus 2050 CS managed harvest), and *H*_*i*,3_’ = 2050 CS managed harvest. Finally, management costs under the CS Scenario are calculated for each country using the same procedure, but where *H*_*i*,3_ is the modeled harvest under the CS scenario, and *H*_*i*,1_’ and *H*_*i*,2_’ = 0. Thus, for each country we have estimates of: the current cost of management, the future cost of management under BAU, the future cost of management if all fisheries not already managed by catch shares adopt CC, and the future cost of management if all fisheries adopt CS.

The final step of this analysis is to compare the incremental cost (of adopting CC or CS, relative to BAU) to the incremental benefits. We use fishery profit as our measure of economic benefit, which was calculated in a previous study examining fishery outcomes under different management regimes [4]. Fishery profit under each management scenario was determined by subtracting fishing costs (management costs are not included in this) from revenue generated by harvest. In that study, profit under catch share management is higher than profit under output control for a number of reasons, including the reduction in fishing costs and the improved product quality often experienced in catch share fisheries. This previous study demonstrated that improved management can lead to greater long-term economic benefit in depleted fisheries by allowing the population to rebuild to more productive levels, as well as in fisheries subject to overfishing by reducing effort in order to maintain or reach healthy population levels. We calculate the incremental benefit of management reform using the outputs of that previous work by subtracting the estimated annual profit in 2050 under BAU from that of the CC and CS management scenarios. We then compare this with the incremental total management cost in 2050 for each country.

To estimate the incremental cost of management, we first calculate the future cost of management for each country in 2050 using Eq 3 for all three future management scenarios. Total harvest in each management category (open access, catch control, and catch share) depends on the management scenario (BAU, CC, or CS Scenario), and was determined in the same previous study described above [4]. Here, we use the harvest results from the previous study as inputs to our cost model. Thus, the incremental cost of management reform is the difference between the total management cost in 2050 for a country under a reform scenario (CC or CS Scenario) and that under BAU. We set the discount rate equal to zero for consistency with the economic benefit data from the previous study.

## Results

We find substantial variation in current management costs across countries (approximately an order of magnitude difference in management cost per MT of harvest) and that the additional costs of upgrading fishery management can be quite substantial (in some countries, this could involve doubling or tripling total management cost). Despite the at times high costs of management upgrades, our overall finding is that in most countries, the benefits of reform substantially outweigh these additional estimated costs. This result holds across a wide range of assumptions and is consistent with empirical data, case studies, and *ad hoc* interviews conducted with fishery managers in countries that have already undergone these transitions.

### CC and CS scenarios compared to BAU, 2050

The additional benefits of CC or CS management reform were compared to the estimated additional costs associated with that reform in the year 2050. To examine outcomes, we calculate the ratio between the expected additional economic benefit (profit) in the year 2050 associated with a reform and the estimated additional cost of management in that year–we refer to this as the benefit-cost ratio (BCR). We calculate a BCR for country *i* under reform scenario *r* (CC and CS scenarios) for the year 2050 using the following equation:

$${BCR}_{r,i}=\frac{\pi_{r,i}-\pi_{BAU,i}}{{TC}_{r,i}-{TC}_{BAU,i}}$$ (6)

where *π*_*r*,*i*_ is total profit in country *i* under reform scenario *r*, *π*_*BAU*,*i*_ is total profit in country *i* under the BAU scenario, *TC*_*r*,*i*_ is the total estimated management cost for country *i* under reform scenario *r*, and *TC*_*BAU*,*i*_ is the total estimated management cost for country *i* under the BAU scenario. A BCR greater than 1 indicates that the benefit is greater than the incremental cost of management in that country, for that particular reform scenario.

**Fig 7:**
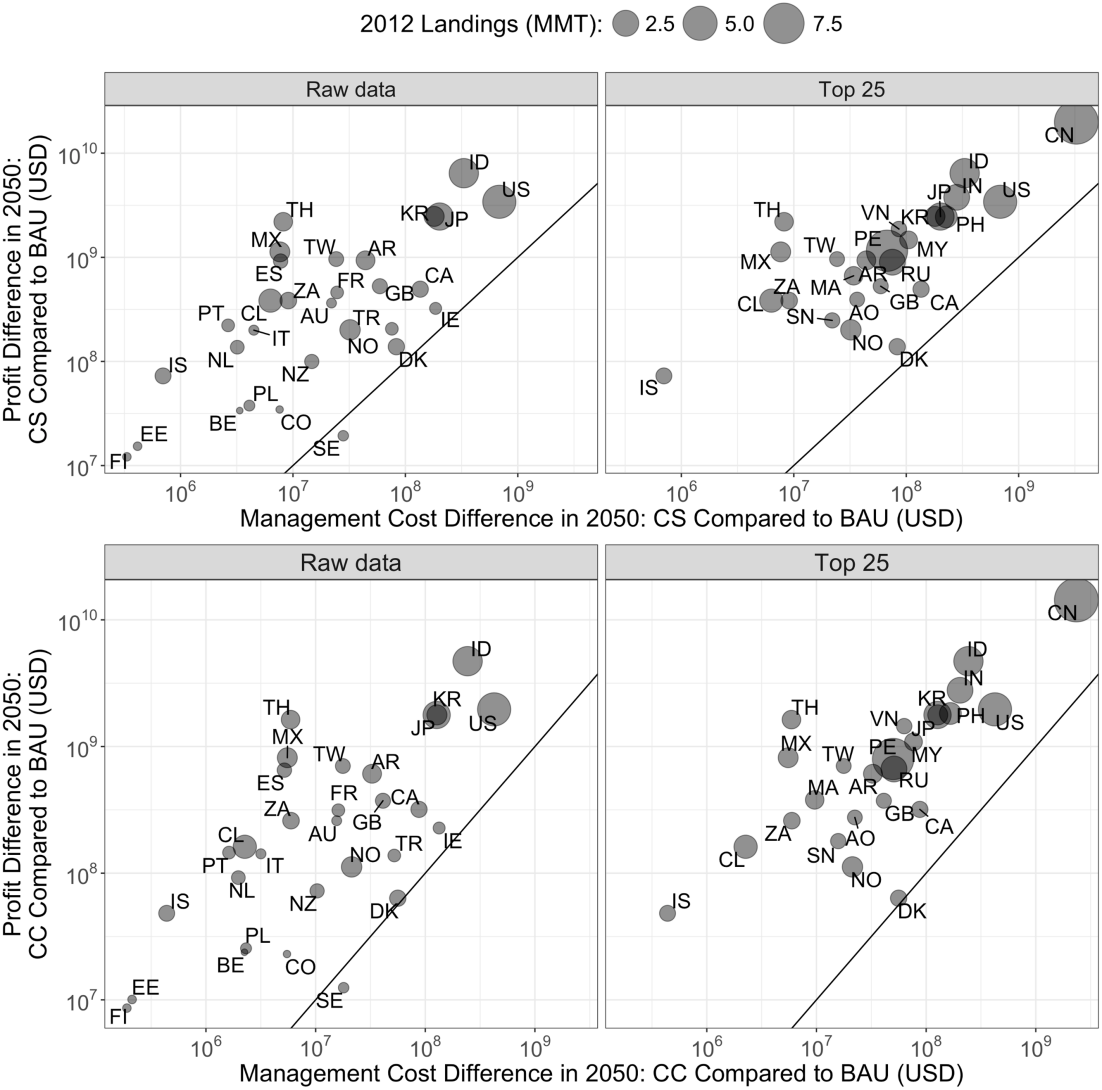
Difference in future profits vs. difference in management cost: CS scenario and CC scenario compared to BAU. Figures on the left include the 30 countries in our management cost database that also have harvest and profit projections from the bioeconomic model. Each country is represented by a single point. The size of the point indicates the size of the fishing sector in that country measured in total harvest (in MT) for 2012. The top panels provide results for CS vs. BAU and the bottom panels provide results for CC vs. BAU. The black diagonal line is a 1:1 line–countries above this line has a benefit-cost ratio greater than 1, and countries below it has a benefit-cost ratio less than 1. Countries are indicated by ISO 3166–1 alpha-2 country codes.

Two findings immediately stand out. The first is that when considering reforming all fisheries in a country to some form of catch share (CS scenario), the cumulative benefits exceed the costs for all 25 of the top fishing nations (Fig 7; see dots above the 1:1 line on the top right). Indeed, the BCRs range from 1.7 up to 268, with a median of about 14 (see S2 Table for BCRs for the top 25 fishing nations). The global BCR (aggregate profit difference compared to aggregate management cost difference) for catch share management is 9.3. Second, all 25 countries still have a BCR greater than 1 with a shift from BAU to CC (Fig 7, bottom right). The global BCR for moving to CC is 9.4. The cost of managing all fisheries in our database under catch share management in 2050 is approximately USD 15.0 billion, which is nearly double the estimated global cost under BAU (USD 8.0 billion) and the 2012 current global management costs (USD 7.6 billion). The global costs under each scenario are listed in Table 2.

**Table 2:** Global management costs. Global management costs to manage the 72.4% of global fish catch represented in our database under different management scenarios.

Management Scenario	Global Cost of Management (billion USD)
2012 Current Cost	7.6
2050 Business as Usual	8.0
2050 Catch Control	12.9
2050 Catch Share	15.0
2050 “Extreme” Cost Scenario	30.91

These results also largely hold for the full set of 30 countries in our management cost database. For all but one country (in Sweden, which has the second highest current cost per MT (USD 544) and already manages nearly all of its fisheries using catch shares or strong output controls (S3 Fig), further management upgrades to either CC or CS result in costs that exceed benefits (Fig 7, left, top and bottom). BCRs for the adoption of CS management range from 0.7 to 268, with a median of 17.6. BCRs for the 30 countries can be found in S3 Table.

As an alternative to our assumption that management cost scales with raw landings, we also repeated the entire analysis assuming that management cost scales with fishery revenue. Under that alternative assumption, BCRs for the top twenty-five countries were qualitatively similar: Under both the CS and CC scenarios all 25 countries have a benefit-cost ratio exceeding one. Under this analysis, BCRs for the adoption of CS management range from 3.3 to 168.1, with a median of about 10.3. For the 30 countries from our management cost database, BCRs equal or exceed one for all countries when adopting CC or CS management. BCRs associated with the adoption of CS management range from 1.0 to 168.1, with a median of 10.4. The global benefit-cost ratios were also similar–scaling costs to landed value results in global BCRs of 7.0 and 10.1 for the adoption of CS and CC management respectively, compared to 9.3 and 9.4 in the alternative analysis that scales costs to landing volumes.

### Extreme cost analysis

Thus far, our results have strongly suggested that even though upgrades in fishery management often entail additional costs, which may be quite substantial, the benefits of these reforms are in most cases economically worthwhile for our examined countries. This analysis has necessarily relied on a number of assumptions.

One test of the robustness of our main result is to make an extreme assumption about the changes in management cost in a country. Here, we undertake the following thought experiment. Suppose we assume that any country wishing to upgrade its management will have to incur costs equal to the average current total cost for the five countries with the most expensive management costs (Ireland, Sweden, Canada, the USA, and Australia), which have an average cost of USD 489 per MT. Only two countries in our cost database have a current cost per MT value greater than this (Fig 2).

**Fig 8:**
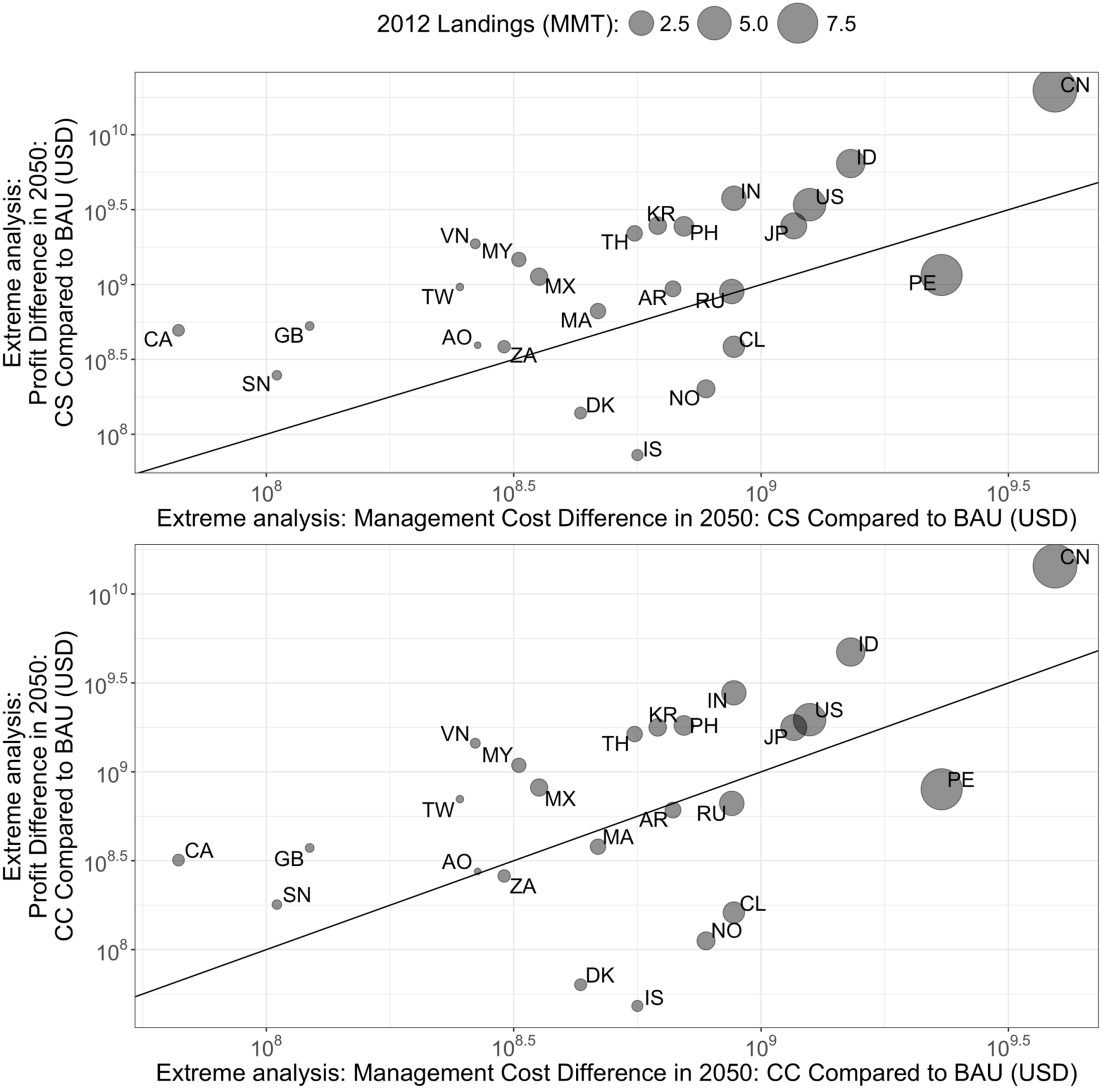
Difference between future profits and management costs for the top 25 fishing nations: CS scenario and CC scenario compared to BAU under the extreme scenario. Countries are indicated by ISO 3166–1 alpha-2 country codes.

We multiply USD 489 by the modeled 2050 harvests under the CS Scenario and CC Scenario to calculate the (extreme version of) the future annual cost of management. The future annual profits from adopting CS are unchanged. Again, the future annual profits and costs of the BAU, CS, and CC Scenarios are compared (Fig 8).

Even under this extreme view of costs, adopting catch shares remains an economically viable option for most of the top 25 fishing nations (Fig 8) and countries in our management cost database. The exceptions are Iceland, Norway, Denmark, Chile, and Peru, which have already largely adopted catch shares (Fig 4), so the additional benefit is not as large. The median BCR for switching to CS under the extreme assumptions is 2.7 for these countries. Even under this extreme scenario, the global BCR is 2.8. The countries with a BCR less than one under the extreme CC scenario are the countries mentioned above, plus Russia, Morocco, South Africa, Argentina, and Angola. The median BCR under this scenario is about 1.7 for these countries, while the global BCR is about 2.0. Under these scenarios, the global cost of management would be USD 30.91 billion.

## Discussion

The overall objective of this work is to develop and implement a framework to estimate whether the benefits of fishery management reform exceed the associated incremental management costs. Our framework for estimating costs of fishery management upgrades incorporates existing data on management costs, survey data on incremental costs, and existing data on current management in each country. We collect, synthesize, and employ these data in a simple model. With that model, we find that the incremental benefits of fishery management reforms are larger than the incremental costs in nearly all countries examined. This result holds when comparing “business as usual” management in a country to adopting some form of rights-based management (or “catch shares”). When considering a less-ambitious move from BAU to catch controls without adopting rights-based approaches, the result still holds.

A key interpretation emerges: while adopting effective catch shares is estimated to entail the largest incremental increases in management cost, it can lead to even more significant increases in economic profit. In fact, some of the experts in our panel suggested that depending on how well fisheries are already managed, the cost of switching to catch share management might even *lower* costs relative to BAU, which would further strengthen our main results. Still, we might expect to see the largest cost increases for countries with relatively poor or ineffective management. If a portion of the expected gains in profit can be captured to pay for the change in management cost, then the policy reform would be win-win. Doing so would require recovering only a small fraction of expected gains in most countries. For countries with BCRs greater than 2 (which is the case for most under both CS and CC management), less than half of the economic gains from management reform would be needed to cover the additional costs of management.

An important question when considering management costs is: Who should pay? It has been argued that because the fishing industry benefits from management services, it should pay the costs associated with that management [19, 3]. Recouping some funds from the fishery to pay for services to the fishery sector, or “cost recovery,” is already employed by a number of countries such as Canada, New Zealand, Iceland, Australia, the United States, Namibia, and Indonesia, and is typically accomplished through fees and taxes within the industry. But in most countries, taxpayers end up paying for these government services. Cost recovery programs in fisheries seek to recover at least a portion of the costs associated with management from those who benefit from the services. These programs also have the potential to minimize government inefficiencies and improve management effectiveness, because fishers will have stronger incentives to employ efficient and cost-effective management once they are responsible for paying for management [20, 21]. Cost recovery also has the potential to increase industry involvement and improve cost accounting [8]. For example, cost recovery in Australia has led to increased industry involvement in management decisions, a more transparent decision-making process, and detailed accounts of management costs [21].

Our main finding is that both CC and CS deliver economic benefits that exceed the costs of their implementation in most countries examined. But which approach gives higher net benefits? To address this question, we calculate for each country the net benefit (total benefit minus total management cost) of adopting CC vs. CS for the top 25 countries. We find that in every country examined, the net benefits are positive (this accords with our results for the benefit-cost ratio). But the more striking result is that for all countries examined, the net benefits of adopting CS outweigh the net benefits of adopting CC. The percentage increase from adopting CS vs. CC ranges from between just 2% (for Iceland, where most fisheries already use CS management) to 58% (for China), with a median across all 25 countries of 44%. Even this might be a conservative result, because we have assumed that securing long-run economic profit is still possible under CC. While output controls alone can be effectively implemented to regulate catch and achieve conservation objectives, there is a strong theoretical argument that they cannot ensure significant long-term profits, because rents will be dissipated by excessive effort on unregulated margins. That is, even if catch is perfectly managed (e.g., to achieve maximum sustainable yield), without a rights-based structure, there may always be margins of adjustment that lead to some rent dissipation. Thus, we regard the CC scenario as an intermediate case between open access and fully rent-capturing catch shares. Even under this interpretation, the aggregate net benefits of adopting CS appears to outweigh the aggregate net benefits of adopting CC for most nations.

While it is clear that management upgrades can lead to economic benefits, the specific interventions and program designs will likely depend on the context of each fishery. While these programs may look quite different, evidence suggests that output controls and catch shares may be effective management options for a range of fishery types. Such programs have been implemented in developed (e.g., Australia, New Zealand, and the United States) and developing nations (e.g., Peru and Mexico) [17]. In addition, upgrades have occurred in a range of fishery types, including large commercial fisheries (e.g., the anchoveta fishery in Peru) and small-scale fisheries (e.g., benthic fisheries in Chile and Mexico) [17]. The species covered in these programs range from short-lived species like anchoveta to long-lived species such as orange roughy in Australia [17].

Because data on management cost are unavailable in many countries, we have had to make a number of assumptions to calculate benefit-cost ratios of improved fishery management. While this suggests that our results are admittedly imprecise, our sensitivity analyses reveal that the magnitude of benefits generated by fishery reform are so large that the costs associated with management upgrades would need to be substantially higher than those included in our database in order to overturn our qualitative findings. As previously discussed, the median benefit per MT from management reform is about 1,515 USD–this is nearly double the most expensive cost estimate in our database (815 USD/MT in Ireland) and about nine times greater than the mean management cost per MT for countries in the database.

Although costs likely do not scale directly with landings under specific management types, case studies broadly support our assumption of increasing costs with management improvements. Canada’s Department of Fisheries Organization (DFO) originally estimated that incorporating an individual quota system in the geoduck fishery (managed by limited entry and a TAC) would cost an additional 250,000 Canadian dollars [22]. This initial estimate was surpassed when the industry, who is responsible for paying for these additional costs, chose to invest additional funding in scientific research [22]. Similarly, costs increased when IFQs were incorporated into the USA’s halibut and sablefish management programs due to the complexity of program rules, increase in number of appeals related to quota allocations, and extended fishing season [23]. Management costs in New Zealand’s inshore fishery was estimated to have increased by 36% after the broad adoption of catch shares [24].

However, annual costs have decreased in the surfclam/ocean quahog program due to the simplicity of the program design, small number of vessels, and population stabilization (reducing the need for extensive management oversight) [23]. This suggest that our approach may have overestimated the costs of reform for some fisheries. However, our approach does not include the fixed cost associated with planning and designing these systems, which can be significant [23]. We test the robustness of our approach by comparing it with an alternative that incorporates an estimate of fixed costs and increasing marginal costs, and find that our approach typically estimates higher management costs (see section B of S2 File and S4 Fig). However, the results from the alternative approach suggests that the early stages of financing could be the most challenging, as costs (fixed and variable) represent a greater proportion of landed value.

Importantly, there are a number of barriers that could prevent countries from adopting fishery reform or prevent reforms from reaching their full potential. Political will and effective governance have been identified as key components that drive fishery reform–therefore, weak or unstable political institutions may prevent the adoption of improved management approaches [25]. Even if reforms are adopted, their success in some contexts may be precluded by corruption or illegal, unreported, and unregulated (IUU) fishing. Corruption in either private or public sectors can undermine management by reducing compliance with regulations. In the Pacific Island region, benefits from fisheries have been limited due to corruption throughout the sector [26]. In addition, illegal, unreported, and unregulated (IUU) fishing can undermine reform initiatives, resulting in unsustainable fishing levels, and reduced harvest and economic value for the nation in which the illegal activity is occurring. Countries with a high prevalence of IUU fishing may need to invest more in enforcement services in order to meet management objectives–this would lead to overall higher management costs and lower overall reform benefits. However, for countries with high benefit-cost ratios, the investment in stronger enforcement programs may still be worth it. Still, for countries with extensive coastlines and limited enforcement capacity, operating an extensive enforcement program may prove infeasible.

### Future work

This study represents a first attempt to estimate future management costs under improved management and compare these costs to the expected benefit of the reform. Mostly due to data limitations, our study relied on a number of assumptions and excluded other potential sources of management costs. Here we discuss a number of ways in which this study could be built upon to further examine the relationship between costs and fisheries management and more precisely estimate management costs associated with reform.

First, while this study focuses on benefits and costs to the fishing industry as a whole, future research might focus on how reforms may affect individual groups or fishers. One concern associated with rights-based management is that it will result in a concentration of quota, creating a situation in which only a small number of (often industrial) participants ultimately have rights to fish. This raises concerns regarding social inequity, especially in areas where small-scale fishers have traditionally had access to resources [27]. However, carefully designed programs (e.g., those that are designed with quota limits or as cooperatives) have the potential to mitigate these issues [27, 28].

We do not model the implications of management reform throughout the supply chain. While processors could benefit from more reliable and higher quality landings, they might also lose bargaining power or suffer other potential outcomes associated with rights-based fishery management [27]. More research is needed to determine the economic implications of improved management on other related sectors.

Second, while this study focused on the annual cost of management after reform has been implemented, studies and interviews indicate that transition costs can be significant. During the transition period, the reform is designed and planned. This stage can be labor intensive and take a substantial amount of time, thus incurring significant fixed costs. In addition, it may require expensive research efforts to guide reform design. Including this expense would capture a more comprehensive cost of fisheries management.

This study does not examine the timing of costs and benefits, but instead focuses on annual indicators for a single year in the future. The costs of reform generally start immediately, while the benefits accrue over time. In fact, depending on the starting biological conditions and the reform implemented, there can be a time period in which the fishery does not generate profits. A future study could examine the timing of benefits, and determine at what point profits from the fishery surpass the cost of immediate design and annual implementation costs. An approach similar to that in McDonald et al. [29] could estimate the time needed for benefits to outweigh costs, as well as the ability for different financing mechanisms to pay for reform. Importantly, this analysis might prove difficult to conduct for an entire country given the data requirements.

Future studies could expand on this work by developing a more precise model for determining changes in management cost. In this study, we chose to scale relative costs of management with management approaches. It is unlikely that the management approach alone affects the cost of management. Complexities within the fishery such as number of species, geographic size, number of participants, and number of landings facilities likely affect the cost of managing it [8, 23]. Similarly, institutional complexities related to rules and regulations such as bycatch regulations, limits on days at sea, gear restrictions, and required reporting and analysis likely influence the costs of administration, research, and enforcement services. For example, a fishery for which a decision has been made to implement onboard observers as a part of the enforcement program may have higher costs than one that does not, regardless of the broader management approach. This approach would require data at a finer resolution than used in the current study. We have only indirectly captured these effects through the country-specific constant, *s*_*i*_. At a much coarser scale is the problem of illegal fishing, which we implicitly fold into monitoring and enforcement costs. But in practice, combating illegal fishing is an effective approach to enhancing legal rent capture in a fishery. Coupling monitoring and enforcement of illegal fishing with catch shares to manage the legal sector may be a highly efficient path forward for many of the world’s fisheries.

**Fig 9:**
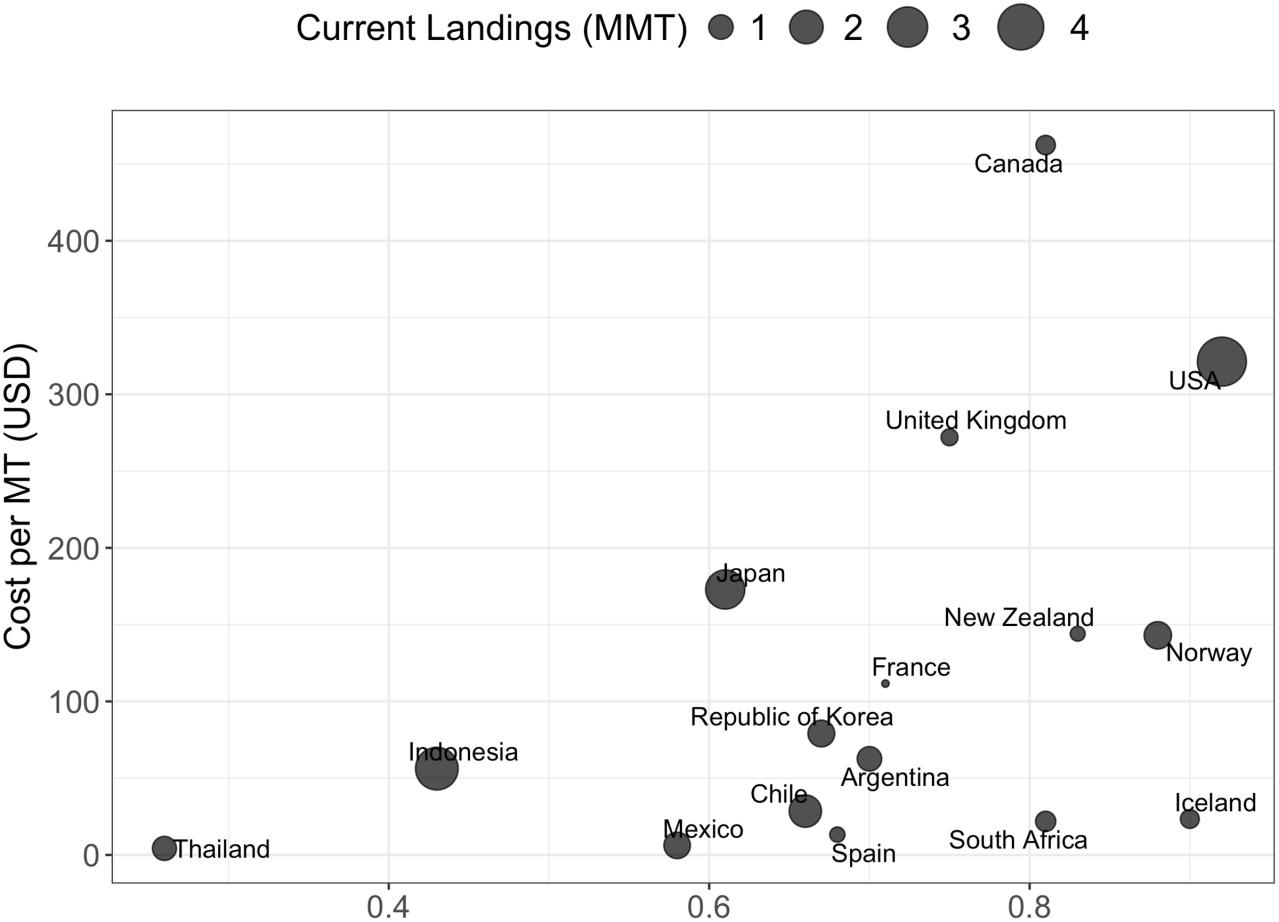
Fisheries Management Index (FMI) scores compared to cost per MT and cost per landed value. Analysis is conducted for countries present in both our management cost database and the FMI study [30].

Future work may also more explicitly account for management efficacy. Although we were able to find management cost data for a number of countries around the world, it is not clear from these numbers alone if the expenditures lead to successful resource management or if the funding is being spent efficiently. This is important, because inefficient spending (a common critique of government spending in general) may make it appear that effective fisheries management is much more expensive than it actually needs to be. To supplement our baseline study, we compared data from our management cost database to country-level Fisheries Management Index (FMI) scores from a recent study [30] in order to investigate the relationship between expenditure and effectiveness. The FMI index ranges from 0 to 1, and was designed to be an indicator of the effectiveness of management institutions at meeting fishery objectives [30]. The comparison suggests that comparatively high expenditures on fishery management are associated with higher FMI scores. Still, the cost per MT of management for countries with FMI scores greater than 0.8 vary substantially, ranging between 22 and 462 USD/MT (Fig 9). More research is needed to tease out the driving forces behind this heterogeneity.

While the country-level approach developed here may help inform decisions at the national level, a fishery level-approach may provide key insights for managers working on the reform of individual fisheries. Individual fisheries may benefit differently from management changes, and effective catch shares will surely require careful design tailored to each fishery. For example, challenges to implementing effective catch share systems might arise in countries that do not measure catch reliably and in fisheries with large and diverse fleets that are hard to monitor. This suggests that other management changes might have to occur before catch shares can be successfully implemented. This approach would require gathering fishery-level data on the cost associated with management attributes specific to fishery type. Improved data on the cost of managing fisheries at both the country and fishery level would facilitate more precise analyses.

The objective of this study is to provide a first attempt at estimating the costs of fishery management upgrades at the country level and ask if the benefits of management upgrades outweigh the costs. While we recognize that our results are imprecise, we have utilized the best available data to address this important question, which we believe is relevant to fishers, policy makers, and managers. Still, as discussed, there are a number of limitations that, if addressed, we believe would improve these estimates. It is our hope that this study will encourage improved data collection regarding the cost of fishery management (at both the fishery and country-levels) and motivate future research on this topic.

## Supporting information

S1 Fig Difference in future profits vs. difference in management cost: CS scenario and OC scenario compared to BAU. Figures on the left include the 30 countries in our management cost database that also have harvest and profit projections from the bioeconomic model. Each country is represented by a single point. The size of the point indicates the size of the fishing sector in that country measured in total harvest (in MT) for 2012. The top panels provide results for CS vs. BAU and the bottom panels provide results for OC vs. BAU. The black diagonal line is a 1:1 line–countries above this line has a benefit-cost ratio greater than 1, and countries below it has a benefit-cost ratio less than 1. Countries are indicated by ISO 3166–1 alpha-2 country codes.
(TIFF)

S2 Fig Survey responses. Survey responses from group of experts.
(TIFF)

S3 Fig Breakdown of current assessments and approaches to management. This figure includes the 30 countries included in the management cost database for which there are future harvest and profit projections from the bioeconomic model. The “Other” category represents landings from fisheries managed under input controls and/or unregulated open access.
(TIFF)

S4 Fig Total cost of management under various cost assumptions for the USA. Total cost of management for the USA under alternative calculation approaches.
(TIFF)

S1 Table Average percentages of total management costs attributed to administration, research, and enforcement services. Values in the second column represent the outcomes when using the most recent administration, research, and enforcement cost reported in each country, while values in the third column represent the outcomes when the mean value of cost in each management category for each country.
(DOCX)

S2 Table Benefit-cost ratio (BCR) results for top 25. Benefit-cost ratios (BCRs) for top 25 countries in terms of 2012 landing volume (MTs).
(DOCX)

S3 Table Benefit-cost ratios (BCRs) for countries in database. Benefit-cost ratios for the 30 countries in the management cost database.
(DOCX)

S1 File Management cost database. Administration, research, and enforcement costs used in this study. This file contains both the most recent cost values and the average cost values for each category in each country.
(CSV)

S2 File Supplementary material and methods. Additional analyses and results.
(PDF)

S3 File Example survey. Survey used to collect information regarding the relevant cost of management from the group of experts.
(XLSX)
