# Supplementary figures and images for: Are fishery management upgrades worth the cost?
Source: PLoS One. 2018 Sep 20;13(9):e0204258. doi: 10.1371/journal.pone.0204258 (PMC6147551; doi:10.1371/journal.pone.0204258)

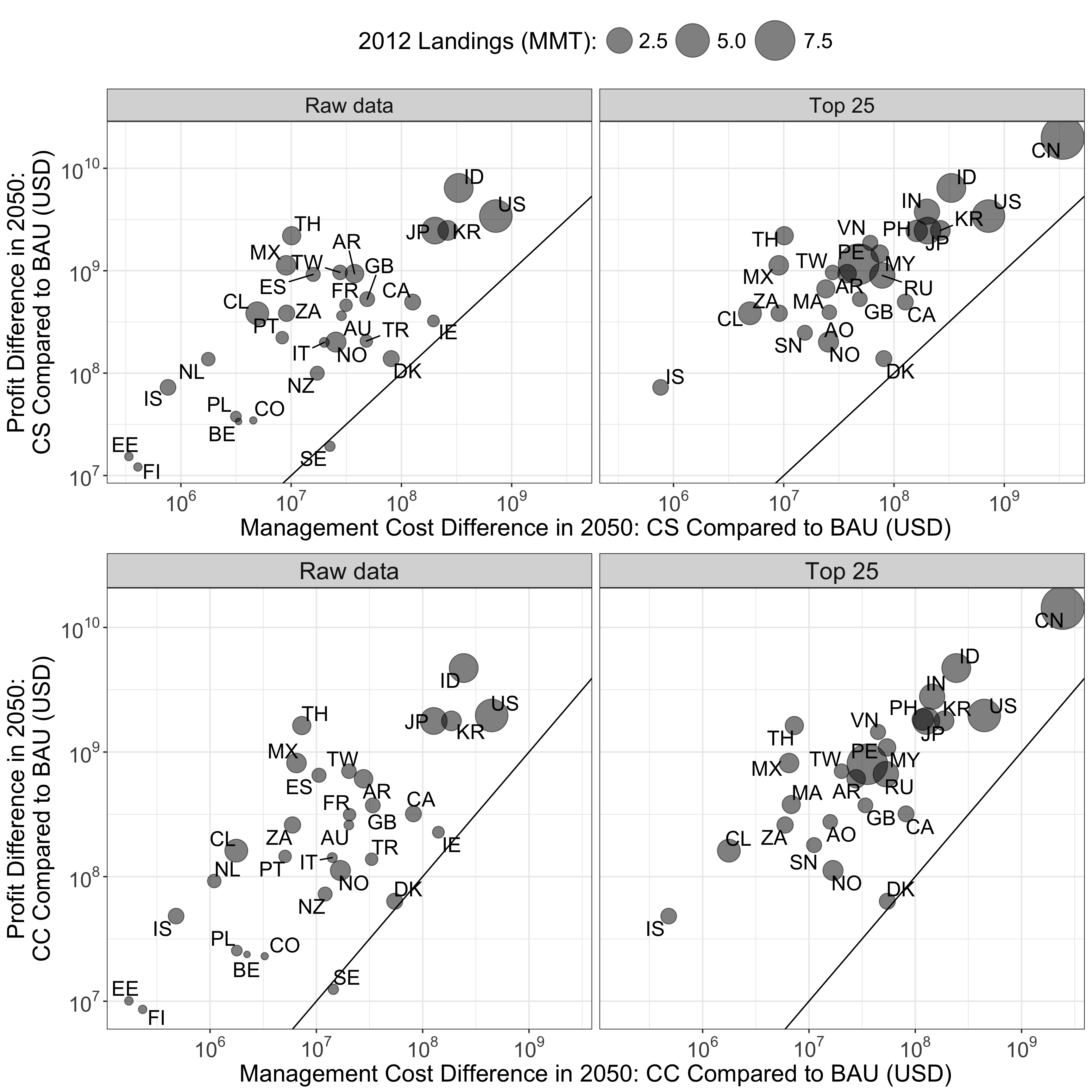

Supplement: S1 Fig — Figures on the left include the 30 countries in our management cost database that also have harvest and profit projections from the bioeconomic model. Each country is represented by a single point. The size of the point indicates the size of the fishing sector in that country measured in total harvest (in MT) for 2012. The top panels provide results for CS vs. BAU and the bottom panels provide results for OC vs. BAU. The black diagonal line is a 1:1 line–countries above this line has a benefit-cost ratio greater than 1, and countries below it has a benefit-cost ratio less than 1. Countries are indicated by ISO 3166–1 alpha-2 country codes. (TIFF) [file pone.0204258.s001.tiff]

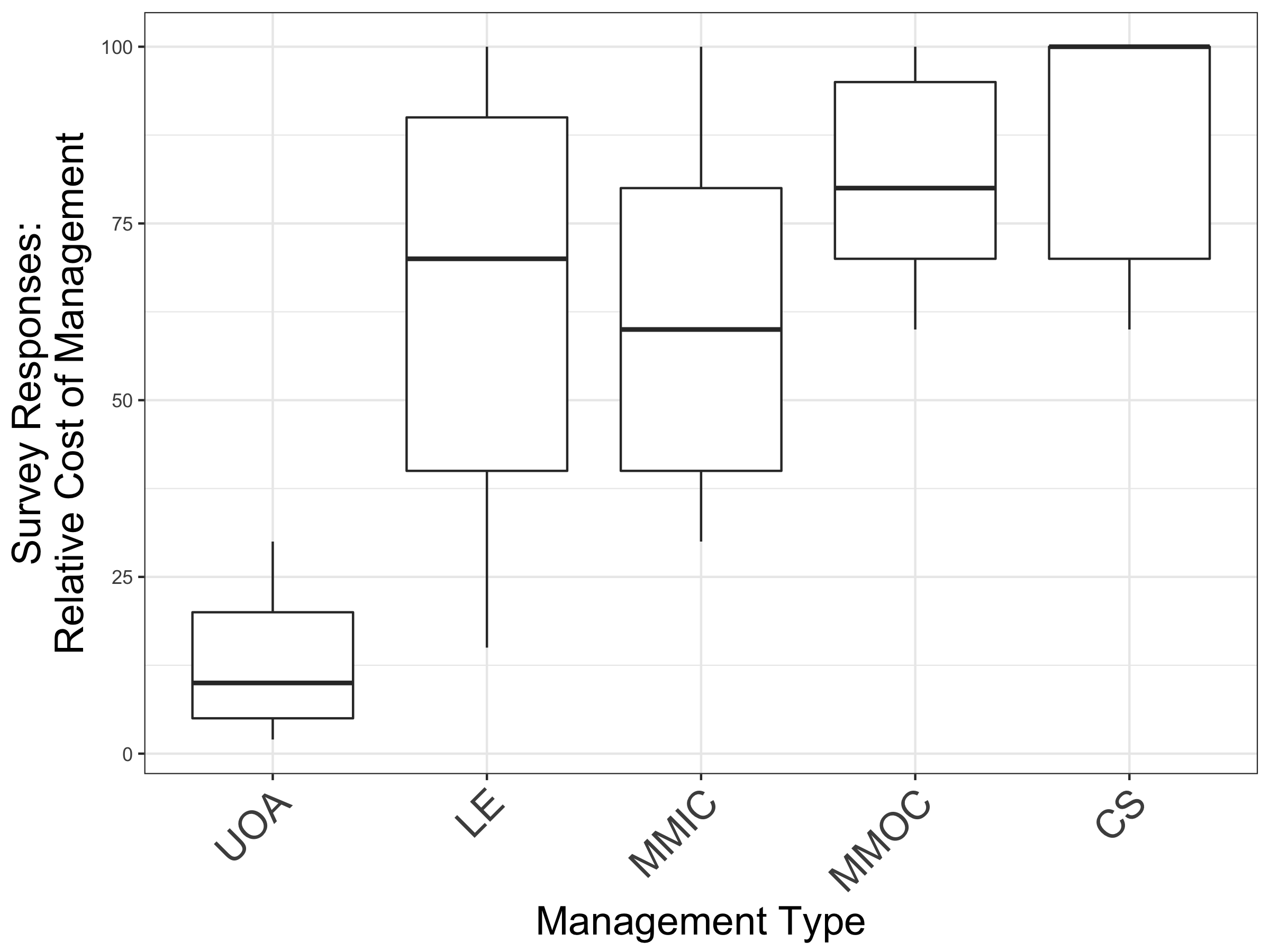

Supplement: S2 Fig — Survey responses from group of experts. (TIFF) [file pone.0204258.s002.tiff]

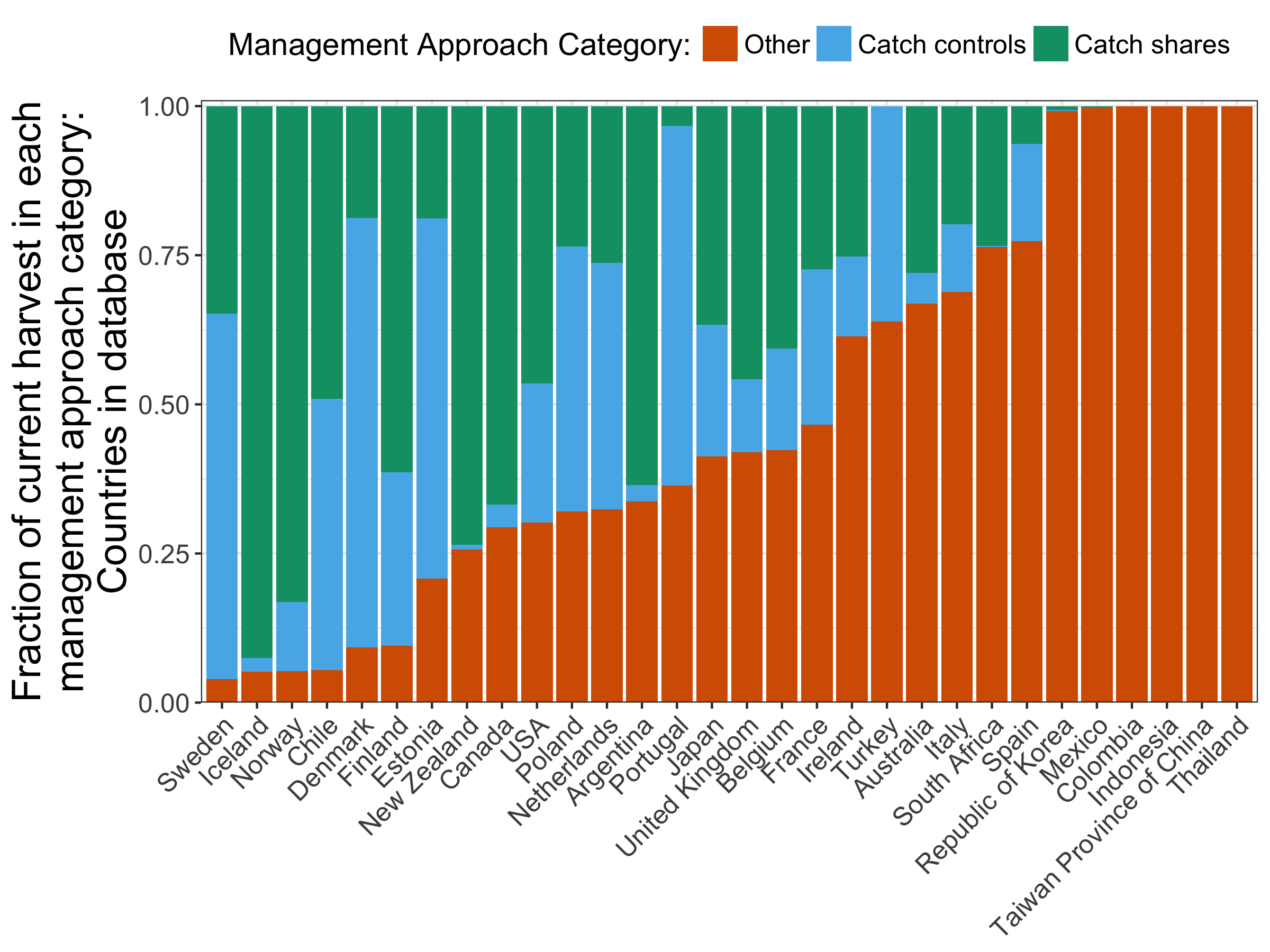

Supplement: S3 Fig — This figure includes the 30 countries included in the management cost database for which there are future harvest and profit projections from the bioeconomic model. The “Other” category represents landings from fisheries managed under input controls and/or unregulated open access. (TIFF) [file pone.0204258.s003.tiff]

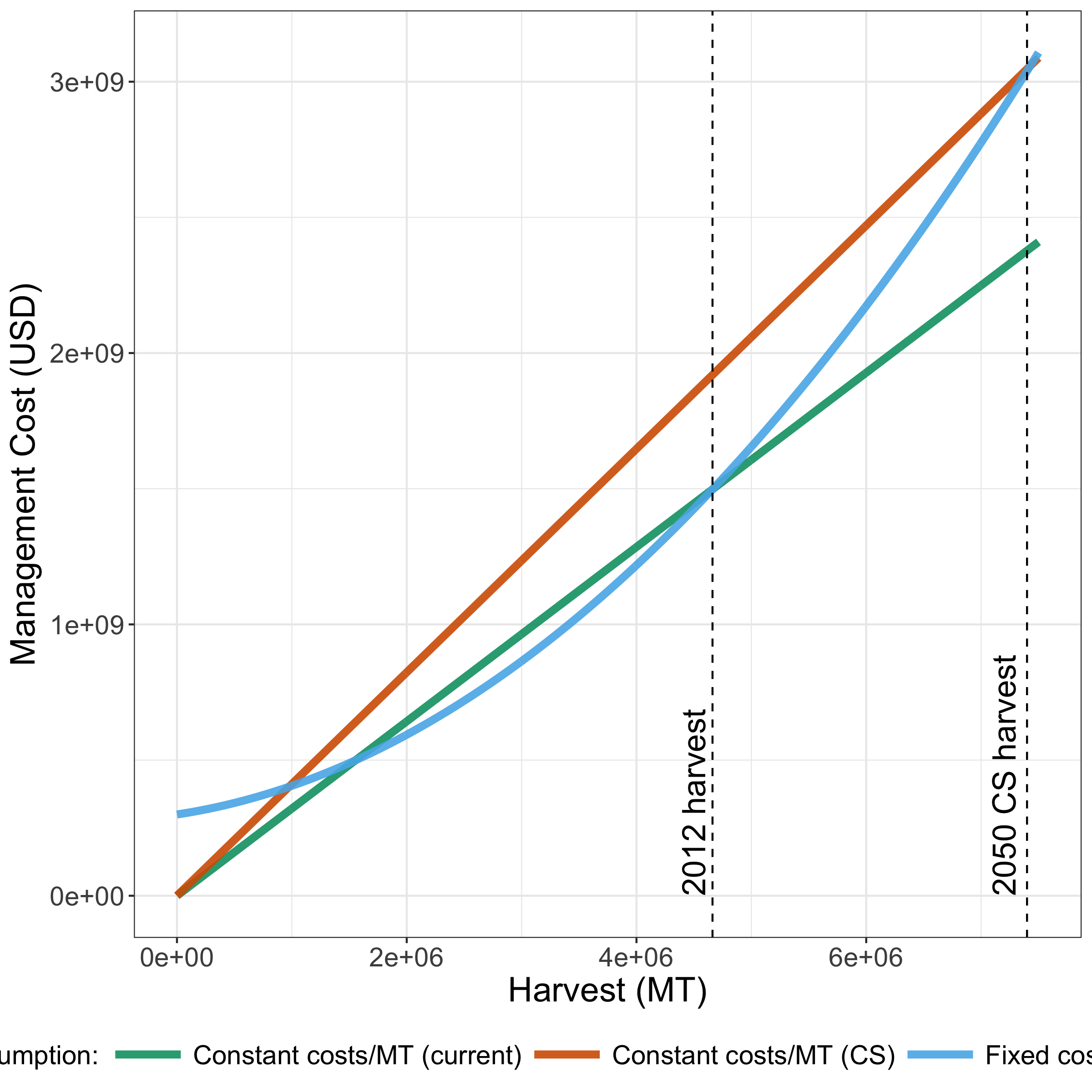

Supplement: S4 Fig — Total cost of management for the USA under alternative calculation approaches. (TIFF) [file pone.0204258.s004.tiff]
