# Supplementary material for: Are fishery management upgrades worth the cost?
Source: PLoS One. 2018 Sep 20;13(9):e0204258. doi: 10.1371/journal.pone.0204258 (PMC6147551; doi:10.1371/journal.pone.0204258)
Supplement: S1 Table — Values in the second column represent the outcomes when using the most recent administration, research, and enforcement cost reported in each country, while values in the third column represent the outcomes when the mean value of cost in each management category for each country. (DOCX) [file pone.0204258.s005.docx]

**S1 Table. Average percentages of total management costs attributed to administration, research, and enforcement services.** Values in the second column represent the outcomes when using the most recent administration, research, and enforcement cost reported in each country, while values in the third column represent the outcomes when the mean value of cost in each management category for each country.

| Management Category | Average percentage across countries using most recent cost data for each country | Average percentage across countries using mean cost data for each country |
| --- | --- | --- |
| Administration | 33% | 34% |
| Research | 28% | 27% |
| Enforcement | 40% | 38% |
