# Supplementary material for: Are fishery management upgrades worth the cost?
Source: PLoS One. 2018 Sep 20;13(9):e0204258. doi: 10.1371/journal.pone.0204258 (PMC6147551; doi:10.1371/journal.pone.0204258)
Supplement: S2 Table — Benefit-cost ratios (BCRs) for top 25 countries in terms of 2012 landing volume (MTs). (DOCX) [file pone.0204258.s006.docx]

**S2 Table. Benefit-cost ratio (BCR) results for top 25.** Benefit-cost ratios (BCRs) for top 25 countries in terms of 2012 landing volume (MTs).

|  |  | Management costs scaled by MT | | Management costs scaled by landed value | |
| --- | --- | --- | --- | --- | --- |
| **Country** | **2012 Landings (MT)** | **BCR - CS** | **BCR - CC** | **BCR - CS** | **BCR - CC** |
| Denmark | 641,506.5 | 1.7 | 1.1 | 3.8 | 4.6 |
| Canada | 627,780.7 | 3.6 | 3.6 | 6.9 | 10.8 |
| USA | 4,661,909.0 | 5.0 | 4.6 | 3.3 | 4.6 |
| China | 9,143,514.2 | 6.1 | 6.1 | 4.8 | 6.8 |
| Norway | 1,277,183.5 | 6.2 | 5.3 | 10.3 | 15.5 |
| United Kingdom | 493,178.3 | 8.9 | 9.1 | 5.3 | 7.5 |
| Angola | 464,426.1 | 10.7 | 12.3 | 7.2 | 12.3 |
| Philippines | 1,541,654.0 | 10.8 | 11.0 | 7.9 | 11.4 |
| Senegal | 517,153.5 | 11.3 | 11.3 | 4.9 | 7.1 |
| Russian Federation | 2,465,313.6 | 11.9 | 13.1 | 8.7 | 12.2 |
| Japan | 2,828,180.9 | 12.2 | 14.0 | 7.1 | 11.3 |
| India | 2,430,416.0 | 13.4 | 13.7 | 10.6 | 15.3 |
| Republic of Korea | 1,217,285.7 | 13.7 | 13.9 | 9.9 | 13.9 |
| Malaysia | 837,884.0 | 14.0 | 14.2 | 10.3 | 14.9 |
| Peru | 7,927,013.2 | 17.2 | 15.9 | 17.3 | 22.7 |
| Indonesia | 3,422,397.0 | 19.4 | 19.4 | 15.1 | 21.3 |
| Morocco | 951,309.2 | 19.5 | 39.4 | 7.8 | 18.4 |
| Argentina | 1,004,535.4 | 21.2 | 18.6 | 17.0 | 21.1 |
| Viet Nam | 532,461.0 | 21.7 | 23.0 | 17.2 | 25.8 |
| Taiwan Province of China | 469,504.0 | 39.7 | 39.6 | 27.9 | 40.0 |
| South Africa | 679,040.1 | 42.3 | 43.6 | 39.0 | 59.3 |
| Chile | 1,845,404.3 | 60.8 | 71.5 | 30.0 | 74.4 |
| Iceland | 594,945.0 | 103.9 | 110.0 | 87.6 | 138.2 |
| Mexico | 1,185,145.0 | 147.6 | 147.9 | 100.4 | 141.1 |
| Thailand | 965,825.0 | 268.0 | 275.9 | 168.1 | 249.1 |
