# Supplementary material for: Are fishery management upgrades worth the cost?
Source: PLoS One. 2018 Sep 20;13(9):e0204258. doi: 10.1371/journal.pone.0204258 (PMC6147551; doi:10.1371/journal.pone.0204258)
Supplement: S3 Table — Benefit-cost ratios for the 30 countries in the management cost database. (DOCX) [file pone.0204258.s007.docx]

**S3 Table. Benefit-cost ratios (BCRs) for countries in database.** Benefit-cost ratios for the 30 countries in the management cost database.

|  |  | Management costs scaled by MT | | Management costs scaled by landed value | |
| --- | --- | --- | --- | --- | --- |
| **Country** | **2012 harvest (MT)** | **BCR - CS** | **BCR - OC** | **BCR - CS** | **BCR OC** |
| Sweden | 122,655.3 | 0.7 | 0.7 | 1.2 | 1.6 |
| Denmark | 641,506.5 | 1.7 | 1.1 | 3.8 | 4.6 |
| Ireland | 211,491.9 | 1.7 | 1.7 | 1.0 | 1.4 |
| Turkey | 264,279.1 | 2.7 | 2.6 | 1.7 | 2.3 |
| Canada | 627,780.7 | 3.6 | 3.6 | 6.9 | 10.8 |
| Colombia | 27,680.0 | 4.5 | 4.2 | 3.1 | 4.6 |
| USA | 4,661,909.0 | 5.0 | 4.6 | 3.3 | 4.6 |
| Norway | 1,277,183.5 | 6.2 | 5.3 | 10.3 | 15.5 |
| New Zealand | 404,132.0 | 6.8 | 7.0 | 10.4 | 15.4 |
| United Kingdom | 493,178.3 | 8.9 | 9.1 | 5.3 | 7.5 |
| Poland | 144,871.7 | 9.2 | 11.0 | 6.2 | 9.6 |
| Belgium | 23,441.4 | 10.0 | 10.6 | 7.4 | 11.2 |
| Japan | 2828,180.9 | 12.2 | 14.0 | 7.1 | 11.3 |
| Republic of Korea | 1,217,285.7 | 13.7 | 13.9 | 9.9 | 13.9 |
| Australia | 91,695.8 | 16.5 | 16.7 | 12.4 | 18.0 |
| France | 264,782.0 | 18.6 | 19.5 | 12.5 | 19.2 |
| Indonesia | 3,422,397.0 | 19.4 | 19.4 | 15.1 | 21.3 |
| Argentina | 1,004,535.4 | 21.2 | 18.6 | 17.0 | 21.1 |
| Finland | 47,177.0 | 36.4 | 45.3 | 6.8 | 10.3 |
| Estonia | 49,016.6 | 36.9 | 47.5 | 8.0 | 11.3 |
| Taiwan Province of China | 469,504.0 | 39.7 | 39.6 | 27.9 | 40.0 |
| South Africa | 679,040.1 | 42.3 | 43.6 | 39.0 | 59.3 |
| Netherlands | 355,661.4 | 42.9 | 46.7 | 26.5 | 41.4 |
| Italy | 101,151.5 | 44.5 | 44.9 | 26.0 | 38.2 |
| Chile | 1,845,404.3 | 60.8 | 71.5 | 30.0 | 74.4 |
| Portugal | 267,079.9 | 83.8 | 89.6 | 34.9 | 51.4 |
| Iceland | 594,945.0 | 103.9 | 110.0 | 87.6 | 138.2 |
| Spain | 418,238.6 | 118.5 | 125.5 | 74.1 | 116.5 |
| Mexico | 1,185,145.0 | 147.6 | 147.9 | 100.4 | 141.1 |
| Thailand | 965,825.0 | 268.0 | 275.9 | 168.1 | 249.1 |
