# Supplementary material for: Are fishery management upgrades worth the cost?
Source: PLoS One. 2018 Sep 20;13(9):e0204258. doi: 10.1371/journal.pone.0204258 (PMC6147551; doi:10.1371/journal.pone.0204258)
Supplement: S2 File — Additional analyses and results. (PDF) [file pone.0204258.s009.pdf]

S2 File. Supplementary material and methods. Additional analyses and results.

Section A: Alternative approach for determining current management costs

In the main text, we use the most recent administration, research, and enforcement cost data available for each country to determine current management costs. Because costs can vary over time, we also complete the entire analysis using the mean administration, research, and enforcement costs over time for each country. The results under this approach are similar to those presented in the main text. First, for the 21 countries that have recorded costs for each of the three management categories, the average percentages of total management costs attributed to administration, research, and enforcement costs are 34%, 27%, and 38% (sum does not equal 100% due to rounding), which is very similar to the result obtained when just using the most recent value reported for each management category (S1 Table).

S1 Table. Average percentages of total management costs attributed to administration, research, and enforcement services. Values in the second column represent the outcomes when using the most recent administration, research, and enforcement cost reported in each country, while values in the third column represent the outcomes when the mean value of cost in each management category for each country.

Management Category	Average percentage across countries using most recent cost data for each country	Average percentage across countries using mean cost data for each country
Administration	33%	34%
Research	28%	27%
Enforcement	40%	38%

Using the mean value of cost in each of the three management categories affects (as opposed to only the most recent value) affects each country's current cost per MT differently. The value obtained using this method ranges between 45% less (Netherlands) and 345% greater (Italy) than those calculated under the method used in the main analysis. On average, cost per MT is 20% greater when using the approach presented here.

The average cost per MT for HDI Group I and Group II countries are similar under both approaches. The average cost per MT for Group I (HDI scores ≥ 0.80) is 184 USD and 191 USD when using the most recent data entries and the mean of data entries, respectively. For Group II, the difference is greater – the value is 81 USD using the most recent data and 58 USD using the average data.

Importantly, the main results are robust to this assumption – the benefit of fishery reform outweighs the cost for nearly all countries examined in our study (S1 Fig)

Section B. Comparison of management cost calculation approaches.

In our analysis, we assumed that all costs are constant, with a cost per MT for each of the three management types. Here, we examine the implications of including fixed costs and incorporating increasing marginal costs into the management cost equation. We use the following equation to model future costs:

$$TC_0 = F + \alpha H_0 + \beta H_0^2 \qquad \text{(S1 Equation)}$$

Where TC_0 is the current (2012) total cost of management, F is the total fixed costs, H_0 is the current (2012) total harvest, α is cost per MT, and β drives the increasing marginal costs. For this analysis, we use the USA as an example, and test the following three approaches for calculating the cost of management:

1. No fixed costs, constant cost per MT equal to current cost per MT (USD 321)
2. No fixed costs, constant cost per MT equal to cost per MT for catch share fisheries (USD 412)
3. Fixed costs, increasing marginal costs

For the third approach, we solve S1 Equation for β and calculate this value using data from our database (current total cost and current total harvest) and the following assumptions:

- F = 20% of current total cost (about 300 million USD)
- α = 20% of current cost per MT (64 USD / MT)

We find that while the approach used in our analysis (option 2) underestimates total costs for comparatively low harvest levels, it largely estimates greater total costs than those under Option 3 (S4 Fig).
